# Farm Animal Veterinarians’ Knowledge and Attitudes toward Antimicrobial Resistance and Antimicrobial Use in the Republic of Serbia

**DOI:** 10.3390/antibiotics11010064

**Published:** 2022-01-05

**Authors:** Jovana Vidović, Dragica Stojanović, Petra Cagnardi, Nebojša Kladar, Olga Horvat, Ivana Ćirković, Katarina Bijelić, Nenad Stojanac, Zorana Kovačević

**Affiliations:** 1 Department of Veterinary Medicine, Faculty of Agriculture, University of Novi Sad, Trg Dositeja Obradovica 8, 21000 Novi Sad, Serbia; jovanavidovic21@gmail.com (J.V.); dragicas@polj.edu.rs (D.S.); zorana.kovacevic@polj.edu.rs (Z.K.); 2 Department of Veterinary Medicine, Università degli Studi di Milano, 20122 Milan, Italy; petra.cagnardi@unimi.it; 3 Department of Pharmacy, Faculty of Medicine, University of Novi Sad, Hajduk Veljkova 3, 21000 Novi Sad, Serbia; NEBOJSA.KLADAR@mf.uns.ac.rs (N.K.); katarina.bijelic@mf.uns.ac.rs (K.B.); 4 Department of Pharmacology Toxicology and Clinical Pharmacology, Faculty of Medicine, University of Novi Sad, Hajduk Veljkova 3, 21000 Novi Sad, Serbia; olga.horvat@mf.uns.ac.rs; 5 Institute of Microbiology and Immunology, Faculty of Medicine, University of Belgrade, Dr Subotica 8, 11000 Belgrade, Serbia; cirkoviciv@yahoo.com

**Keywords:** antimicrobial stewardship, antimicrobial resistance, antimicrobial therapy, veterinarians, mastitis, farm animals

## Abstract

Antimicrobial resistance (AMR) is considered one of the most prevalent global health issues in both veterinarian and human medicine. This complex problem requires a “One Health” approach with the cooperation of all healthcare sectors, as well as agriculture, finance, and consumers. We conducted a survey with the objective to assess the knowledge and attitudes of farm animal veterinarians toward AMR and antimicrobial use in the Republic of Serbia with a small focus on mastitis therapy. A total of 110 respondents completed the questionnaire, which represents a response rate of 27.3%. The majority of our respondents (*n* = 102, 92.7%) completely agreed that AMR currently represents severe concern in the health sector. Unfortunately, less than one-third (*n* = 34, 30.9%) of the respondents had only heard about antimicrobial stewardship. Participants showed a positive attitude toward prudent antimicrobial use and were open to solutions to the AMR crisis. We noticed a certain gap between farm veterinarians’ desire to improve and perform better in daily practice, while at the same time feeling like they did not have enough guidance, help, and resources.

## 1. Introduction

Antimicrobial resistance (AMR) is a global health threat that continues to rise in both veterinary and human medicine [1]. The causal relationship between AMR and misuse and/or excessive antimicrobial usage has been strongly established at this point [2,3]. Even though failure in therapeutic procedures requiring antimicrobials in veterinary medicine due to AMR is far less common than in human medicine, there have been well-documented cases of resistant bacteria in animals, including their transmission to humans and vice versa [4,5,6,7,8,9]. This means that AMR spread is a complex issue since there are many ways in which AMR can be transferred between humans and animals, via close contact, through the food chain, or indirectly via the environment [10,11], making a collaborative approach to AMR under the principles of “One Health” a necessity [12,13]. This is especially important as the antimicrobials used in human medicine are to some extent the same as those used in veterinary medicine [14,15], and antibiotics with critical importance for human medicine must be avoided whenever possible.

Extensive research has been done in human medicine regarding the misuse of antimicrobials [16,17], while, in veterinary medicine, this type of research has only recently gained some traction. Veterinary professionals differ in their approach to the subject depending on the type of sector they belong to, i.e., companion animals, livestock, or wild animals. All of them play an important role in the rational use of antibiotics (AMU). However, farm veterinarians occupy a specific part in this system, making their knowledge and attitude toward AMR and AMU essential for the scope of the problem itself. Furthermore, sales of veterinary antimicrobials in 31 European countries in 2018 have shown that the overall AMU in production animals is substantially higher compared to companion animals [18].

It is very important to obtain a better understanding of veterinarians’ prescribing habits since they prescribe antimicrobials for prophylactic, metaphylactic, or therapeutic purposes [19]. Even though this is a banned practice in Europe, in some countries across the world, antimicrobials are also used as growth promotors [20]. It is necessary to preserve and extend the effectiveness of known and available antimicrobial drugs with prudent AMU. This is why we should strive to minimize the potential for AMR, while maximizing the antimicrobial effect, especially with high-quality antimicrobial stewardship (AMS) programs.

AMS describes all strategies and actions that can help the optimization and the rational use of antimicrobials [21]. Since these actions, when properly applied, can help reduce the spread of AMR, it is important to guide stewardship in the veterinary sector. This can be implemented through monitoring of farm-level antimicrobial use [22] or increased biosecurity [23,24] and AMR monitoring [25].

Farm animals are exposed to considerable quantities of antimicrobials [26], especially in bovine mastitis treatment, where it can lead to the development of AMR and a decrease in successful antibiotic therapy [27]. Moreover, they can act as an important reservoir of AMR genes, which could be transmitted to humans through the food chain, direct animal contact, and the environment [28].

However, the concerns about AMR require the dairy industry to reduce the use of antibiotics. Since, in the Republic of Serbia, there are no developed guidelines for the rational AMU in veterinary medicine, there is an urgent need for understanding the attitude and knowledge of veterinarians toward primarily AMR, but also AMU and AMS, as a crucial step for the design of strategies and interventions to combat this public health threat [28]. Furthermore, the “One Health” approach is severely underdeveloped.

In the Republic of Serbia, total AMU in human medicine was found to be well above the European average [29,30], while the data from veterinary medicine cannot be compared with Europe. Actually, the Medicines and Medical Devices Agency of Serbia collects and processes data from stakeholders who are obliged to keep records on the type and quantity of veterinary medicinal products sold in the Republic of Serbia [31]. These data are presented with ATC codes for drugs, in kg of active substance [32], without an established information database and possibilities for more precise analysis, making it impossible to provide more detailed insight (AMU by species or categories of animals) into the consumption volume, regardless of formulation and package size [33]. Additionally, a highly required extensive research in accordance with the requirements of European Union (EU) Decision 2020/1729 [34] has never been conducted in the Republic of Serbia; consequently, there are no relevant data on the prevalence of antimicrobial resistance or the possibility of determining indicators for monitoring AMR of bacteria in animals. This indicates that the situation with AMR in the veterinary sector is predominantly reliant on veterinarians’ knowledge, consciousness, and attitudes.

Considering all the factors mentioned, and the fact that, to our knowledge, there have not been similar articles published, the objective of this study was to assess attitudes and knowledge regarding AMR and AMU among farm animal veterinarians in Serbia.

## 2. Results

### 2.1. Sociodemographic Data

A total of 110 farm animal veterinarians participated in the survey. A summary of sociodemographic data is presented in Table 1. The majority of respondents were males (*n* = 92, 83.6%), while females made up 16.4% (*n* = 18) of respondents. There were 25.5% of respondents who were 25–34 years old and only one participant (0.1%) over 65 years old. Participants were of different educational levels, with 76 (69.1%) having a first degree, six (5.5%) having a master’s degree, 14 (12.7%) having a doctorate, and 14 (12.7%) having a specialist degree. Eighty-four respondents (76.4%) worked in the private sector, while 23.6% worked in the state sector. Of the 110 veterinarians, more than half had already worked in practice for 6–15 years (*n* = 56, 50.9%) and one-third (*n* = 37, 33.6%) had more than 15 years of work experience.

### 2.2. Significance of Bacterial Resistance to Antibiotics

A great majority of respondents (*n* = 80, 72.7%) had received some sort of educational classes regarding AMU and/or AMR in the last 3 years, while 29 of them (26.4%) had no education on the subject at all, and one participant (0.9%) had no recollection of either happening. Many respondents used foreign or domestic guidelines when prescribing antibiotic therapy with 32 (29.1%) using them often, 39 (35.5%) using them moderately, and 25 (22.7%) using them rarely. Only six (5.5%) participants did not use the guidelines, while eight (7.2%) considered that there were no good quality guidelines available. Even so, 97 (88.2%) respondents thought that there was a need for more local guidelines, and only seven (6.4%) thought that there was no need for them.

The respondents encountered bacterial infections resistant to antibiotic treatments in their daily (*n* = 5, 4.5%), weekly (*n* = 28, 25.5%), or monthly (*n* = 31, 28.2%) practice, while 44 (40%) of them experienced those situations rarely and two (1.8%) experienced them never (Table 2).

Twenty percent (*n* = 22) of respondents indicated that they had protocols for prescribing antibiotics in their practice, while 80% (*n* = 88) did not. However, 87.3% (*n* = 96) considered that those protocols should exist, while 12.7% (*n* = 14) disagreed. A majority (*n* = 79, 71.8%) kept records of AMU, and some (*n* = 31, 28.2%) did not. Regarding prescribing antibiotics outside of suggested clinical indications for their usage, respondents did it often (*n* = 12, 10.9%), moderately (*n* = 28, 25.5%), rarely (*n* = 47, 42.7%), or never (*n* = 23, 20.9%). When asked to which extent the use of antibiotics by unqualified people negatively impacts AMR, 91.8% (*n* = 101) of participants considered the impact to be significant, 7.3% (*n* = 8) considered it moderate, and 0.9% (*n* = 1) thought that there was no impact (Table 2).

When asked about antimicrobial stewardship, less than one-third (*n* = 34, 30.9%) of respondents knew what the term meant, while more than two-thirds (*n* = 76, 69.1%) had never heard about it (Table 2). We provided the definition of AMS in the questionnaire before asking the respondents about the potential influence of implementing AMS guidelines on various important sectors of the current AMR situation. Results suggest that only 11.2% (*n* = 21) of respondents thought that the implementation of AMS would not lead to any significant changes, while the rest assumed that it would lead to a reduction in AMR in humans and animals and an increase in the responsible use of antibiotics (Table 3).

A large number of participants considered animal products (*n* = 68, 38.9%) and contact with animals (*n* = 33, 18.9%) to be the main sources of AMR in humans (Table 4).

Participants were further asked about their opinion on the impact level of different sectors on the development and spread of AMR. The defined sectors were “farm hygiene” as a representation of biosecurity, “rational prescribing of AB” as a representation of veterinary influence, and “application of AB therapy by animal owners” as a representation of therapy application of antimicrobials by the animal owners. They thought that all of the sectors had a great impact on AMR; however, “application of AB therapy by animal owners” had the biggest impact values (*n* = 79, 71.8%), followed by “rational prescribing of AB” (*n* = 77, 70%) and “farm hygiene” (*n* = 47, 42.7%) (Table 5).

When presented with 11 alternatives to antibiotics, participants were mostly familiar with probiotics (*n* = 87, 20.5%) and vaccines (*n* = 80, 18.9%), and least familiar with phage therapy (*n* = 9, 2.1%) and nanoparticles (*n* = 7, 1.7%) (Table 6).

With respect to the AB therapy itself and its influence on the development of AMR, respondents considered that “excessive use of AB” (*n* = 103, 93.6%), “AB use without clear indications” (*n* = 81, 73.6%), “wrong therapy length” (*n* = 74, 67.3%), and “low dosage of AB” (*n* = 69, 62.7%) had a great impact on this issue (Table 7).

### 2.3. Veterinarians’ Prescribing Habits

When asked about the reason for prescribing antibiotics without clear indication, it seems that the participants did it rather frequently because of the cost of tests (*n* = 67, 23.8%) and the lack of quick diagnostic tests (*n* = 61, 21.6%), followed by pressure from animal owners (*n* = 47, 16.7%), prescribing habits (*n* = 38, 13.5%), the lack of clear guidelines for certain diseases (*n* = 35, 12.4%), and insufficient education of veterinarians (*n* = 34, 12.1%) (Table 8).

Amongst the factors influencing them when prescribing AB, the participants pointed to clinical symptoms (*n* = 78, 70.9%), antibiograms (*n* = 72, 65.4%), and milk withholding period for drugs (*n* = 69, 62.7%) as very important. The spread of AMR amongst people and animals also had a high status with 51.8% (*n* = 57) and 56.4% (*n* = 62) of respondents, respectively, rating them as very important (Table 9).

Regarding antibiotic prescribing habits, it seems that the participants mostly (*n* = 106, 77.4%) prescribed antibiotics exclusively for therapeutic purposes (Table 10).

**Figure 1:**
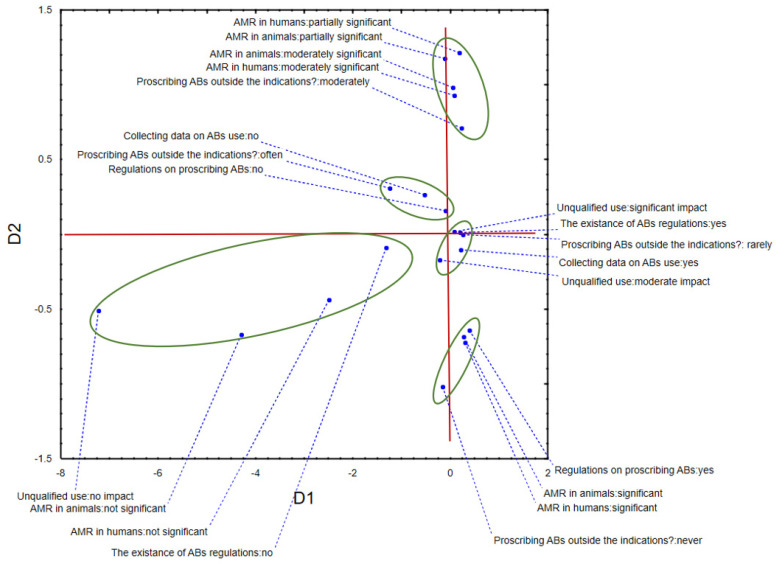
The position of respondents’ answers in space defined by the first two multiple correspondence axes—rational prescribing of antibiotics.

The application of multiple correspondence analysis (MCA) to the data describing respondents’ opinions on rationally prescribing antibiotics shows that the first two dimensions (Ds) described more than 30% of data inertia (D1 = 17.00%, D2, 13,66%). The position of recorded answers in the space defined by D1 and D2 revealed a separative grouping, mostly in the space defined by D2. Specifically, we can notice that respondents that considered AMR an important factor when prescribing antibiotics also never prescribed them without clear indication (sensitivity tests, i.e., antibiograms)—negative part of D2. Furthermore, in the negative part of D1, respondents that did not take AMR into consideration and did not think that handling of antibiotics by unqualified people could be detrimental for AMR were separately grouped, in addition to veterinarians that often prescribed antibiotics outside their indications and did not have prescribing protocols or collected data on AMU (Figure 1). The positive space of D1 was reserved for veterinarians who shared the awareness of antimicrobial resistance existence.

### 2.4. Attitudes toward AMR

Participants acknowledged that AMR is an emerging problem in both human and veterinary medicine today with 102 (92.7%) completely agreeing, no participants completely disagreeing, and only two (1.8%) slightly disagreeing with this. They also mostly completely agreed (*n* = 99, 90.0%) that it will become an even bigger problem in the near future if we do not act rapidly. Slightly less than half of the respondents (*n* = 51, 46.4%) strongly agreed that they contributed to the spread of AMR, and 85 (77.3%) of them were completely open to new solutions in the forms of alternatives to antibiotics if clinically proven to be effective. The respondents also had strong opinions on over-the-counter antibiotics, with 79.1% (*n* = 87) of them completely agreeing it should be a prohibited practice (Table 11).

**Figure 2:**
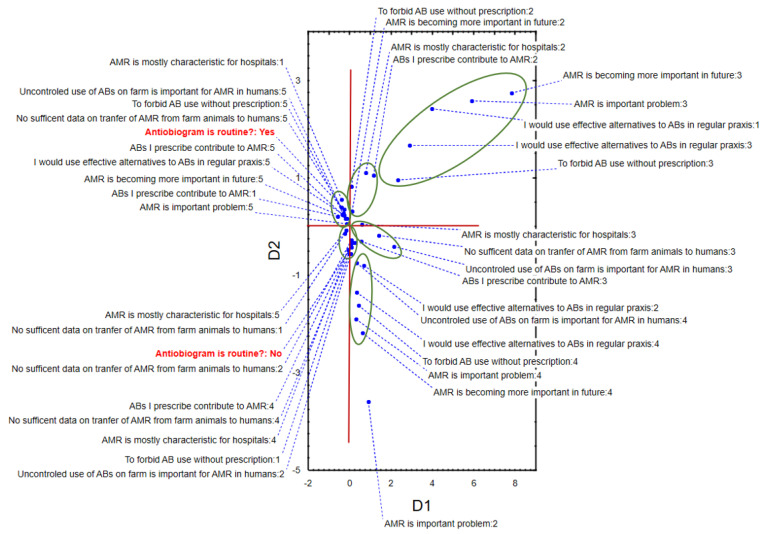
The position of respondents’ answers in space defined by the first two multiple correspondence axes—knowledge on AMR and antibiotic treatment of mastitis in cows.

The application of MCA to respondents’ opinions regarding knowledge on AMR and antibiotic treatment of mastitis in cows shows that the first two dimensions described around 18% of data variability (D1 = 10.19%, D2 = 8.22%). The position of the recorded answers in the space defined by D1 and D2 highlights the grouping of veterinarians that considered AMR a big issue currently and even worse in the near future, and that performed antibiograms routinely—in the negative part of D1 and positive part of D2. Furthermore, the same respondents shared the opinion of prohibiting over-the-counter use of antibiotics and also considered that the uncontrolled use of antibiotics on farms is important for AMR in humans, but did not think that we have sufficient data on the transmission of AMR from farm animals to humans. On the other hand, respondents that considered AMR exclusively a hospital setting issue did not perform antibiograms routinely and were localized in the negative part of D1, as well as the negative part of D2. Moreover, they did not consider that using antibiotics without prescription is dangerous, or that uncontrolled use of antimicrobials on farms can also increase AMR in humans (Figure 2).

### 2.5. Cow Mastitis Therapy

Participants were asked to choose the three most frequently used antibiotics, out of 15 suggested, in cow mastitis therapy. The most frequently used were enrofloxacin (*n* = 56, 17.4%) and amoxicillin (*n* = 48, 14.9%), followed by amoxicillin + clavulanic acid (*n* = 48, 14.9%). The least used antibiotics were erythromycin (*n* = 4, 1.2%), lincomycin (*n* = 3, 0.9%), and novobiocin (*n* = 2, 0.6%) (Table 12).

Respondents’ prescribing habits regarding cow mastitis therapy were influenced by many factors, but mostly by their professional experience and knowledge of clinical symptoms (*n* = 91, 60.6%). Furthermore, only 16.7% (*n* = 25) of respondents used an antibiogram when prescribing antibiotics in therapy (Table 13).

## 3. Discussion

To the best of our knowledge, there are no published articles about veterinarians’ attitudes toward and knowledge on AMR, AMU, and AMS in the Republic of Serbia. So far published data regarding this issue were focused on veterinary students’ knowledge and comprehension [35].

Hence, this study aimed to evaluate farm animal veterinarians’ knowledge and attitudes toward AMR and AMU in the Republic of Serbia. The majority of participants included in the study (42.7%) were 35–44 years old, with 84.5% having at least 6 years of experience working in the practice.

As mentioned before, AMR represents a global health threat, and a large number of organizations are working on decreasing drug resistance worldwide. One established method to combat AMR is the implementation of good practice AMU guidelines [35,36]. As in other similar studies [36,37], we found that many of our respondents used foreign or domestic guidelines when prescribing antibiotic therapy. Since most of them considered that there is a requirement for publishing more local guidelines, this depicts a strongly positive attitude toward improving their knowledge and taking their role in the system seriously; however, they might feel like they do not have enough support and guidance in their practice. This is something that should be addressed in the future national activities as there is evidence that compliance with AMU guidelines might reduce the overall AMU [38]. Tailormade interventions and a close cooperation with the herd veterinarian were shown to be the key determinants in a successful response to the challenges of AMU reduction [39].

Unfortunately, less than one-third of our participants (30.9%) were previously familiar with the term “antimicrobial stewardship”, which shows that there is still a lot of work to be done in promoting this concept and raising awareness in the veterinary sector to facilitate the implementation of AMS strategies. This can be explained by it being a relatively new term in the veterinary profession, and veterinarians might be familiar with its principles only in theory. Contrary to our findings, 63% of veterinarians from Nigeria were familiar with the AMS term [40], while, in the same country, another survey showed that only 17% of them had heard about AMS [41]. The first result is perhaps more representative of the situation since they targeted the whole country, while the second one targeted one state.

Regrettably, AMS programs are yet to be widely applied in veterinary medicine. In line with the global WHO action plan [42], the Republic of Serbia has adopted a national strategy aimed at improving AMS programs [43], but there have not been any significant steps taken for the realization of this strategy. Research in Australia showed that some of the key barriers for this can be AMS governance structures, client expectations, competition between practices, cost of microbiological testing, and lack of access to education, training, and AMS resources [44]. Recent data imply that European veterinary students, including students from Serbia, are aware of this issue and feel the need for better and improved education on AMS and AMR [45].

A very low percentage (20%) of our respondents had protocols for prescribing antibiotics in their practices compared to more than half of Nigerian respondents [40] and more than half of veterinarians’ clients from international research [46]. Nevertheless, 87.3% of our veterinarians promisingly thought that those protocols should exist. This once again highlights the dichotomy between commendatory veterinarians’ attitudes and the sometimes lacking reality of practice.

Regarding the strong correlation between AMU and the spread and development of AMR [11], it is important to educate veterinarians about responsible and prudent AMU as a basis for AMR reduction. Hence, monitoring AMU at the farm level could be one of the crucial steps. For this reason, it is very favorable that more than two-thirds (71.8%) of our participants kept records of AMU in their practice. In the United States, data on this issue are not consistent. In some parts of the country, e.g., at a veterinary teaching hospital, patient records were well kept [47], whereas, in the northeastern part of the country, clinicians frequently prescribed antimicrobials without medical records [48]. Furthermore, about 70% of veterinarians in our survey found that AMU and rationally prescribing antimicrobials could have a great impact on the development and spread of AMR. On the contrary, veterinary students from Croatia and Serbia showed insufficient awareness of veterinary medicine’s AMU contribution to overall AMR, since only 56.8% chose a strong contribution as the answer [35]. This indicates that some considerations on this issue in the veterinary sector exist, but there is still a lot more that can be done through continuous education of current veterinarians. Additionally, our results are more promising compared to a report from an Australian study, where over 50% of veterinarians found AMU to have a moderate influence on AMR [44]. Furthermore, significant facilitators to veterinarians’ prudent antimicrobial prescribing in the cattle and pig livestock sector included education, veterinarians’ positive attitudes toward AMU reduction, and diagnosis [49].

A study conducted in the Netherlands suggested that veterinarians with a positive attitude and sufficient knowledge of AMR can have a positive impact on AMU [50]. However, our study discovered a large gap in the veterinarians’ knowledge regarding AMR etiology, since data show that, although resistant bacteria AMR can be transmitted via contact with other people [51] and animals [52,53], this was generally not recognized among the respondents. This shows that a lot of effort has to be put into teaching current veterinarians about the AMR issue throughout different educational campaigns.

When it comes to alternatives to antibiotics, the veterinarians in our study were most familiar with probiotics (20.5%) and vaccines (18.9%), while phage therapy (2.1%) and nanoparticles (1.7%) were less known to them. Our results are similar to a study where vaccines for the prevention and control of calf scours were recommended on 24% of dairy farms in Italy [54]. Contrary to this, a similar study performed in the same country revealed that 64.5% of cattle veterinarians suggested/prescribed alternative approaches to the use of antimicrobials [55]. Promisingly, when it comes to antibiotic therapy’s influence on the development of AMR, the majority of respondents in our study were aware that the excessive use of antibiotics, low therapy dosage of antibiotics, antibiotic use without clear indications (antibiograms), and inadequate therapy length had a great impact. Furthermore, a great majority of our respondents (77.3%) showed positive attitudes and openness to using antibiotic alternatives if proven to be successful in clinical practice. This is important since there is a global strategy [56] aimed at reducing AMR, which can be implemented by the development of new antibiotics [57], a seemingly difficult and slow task [58], or the application of safe and efficient alternatives to antibiotics [59,60].

A systematic review article of 34 studies published in 2021 showed that the most important factors influencing veterinary health professionals’ selection of an antibiotic in therapy are sociodemographic characteristics, influenced by different attitudes, business factors, and complacency, as well as owner-related factors, such as lack of awareness and demand for antibiotics [61]. Another study that included 25 European countries indicated the following factors as important for selection of an antibiotics: sensitivity test results (antibiograms), their own experience, the risk of developing AMR, and ease of administration [62]. In line with these European countries, in our research, veterinarians indicated antibiograms (65.4%) and concern over AMR development in animals (56.4%) and people (51.8%) as very important in the decision-making process. Similarly to our results, 75.8% of veterinarians in the USA reported antibiograms as an extremely important factor [47].

Our findings also suggest a correlation between veterinarians that perform antibiogram tests routinely and the awareness level of how uncontrolled AMU influences AMR development. Forty-nine percent of our respondents performed antibiograms routinely, which is a higher level compared to the 38% of European veterinarians [62] and lower than recorded for small animal veterinarians in South Africa (71.8%) [63]. Failure in initial therapy usually encourages sensitivity testing to become a part of diagnosis [64]. Furthermore, not much is known about the decision making concerning antibiograms [62]. In the bovine, porcine, and equine sectors, the financial aspect (linked to sampling or analysis) was one of the biggest hurdles to the use of antibiograms [65]

Even though the respondents primarily prescribed antibiotics for therapeutic purposes, there was still a presence of prophylactic and metaphylactic use of these drugs that may have consequences for the increase in AMR [66]. Similar practices have been reported across other European countries [55,67,68]. However, the level of respondents that conducted AST tests routinely was significantly low compared to other studies [69], which can be explained by our respondents being influenced mostly by owners’ financial situation/cost of tests (23.8%) and the lack of quick diagnostic tests (21.6%) when prescribing antibiotics without sensitivity tests.

Almost all of the surveyed veterinarians (92.7%) completely agreed that AMR is an important issue in both human and veterinary medicine, corresponding with most Bhutan veterinarians (96%) [36]. Furthermore, participants in our study believed that AMR will become a much more serious problem in the near future if we do not act rapidly in the present (90.0%), which is similar to the opinion shared by veterinarians in Australia (91.8%) [70]. On the other hand, only half of them were aware that the antibiotics they prescribe contribute to the problem of AMR. Likewise, in Australia, over 60% of veterinarians indicated that their AMU only had a minimal contribution to AMR [44], whereas, in Kentucky, USA, most veterinarians (93%) indicated that improper AMU contributed to selection for AMR [71]. The lack of awareness regarding this subject can be dangerous since the absence of personal responsibility for problematic outcomes reduces the chances of change [72]. This is of tremendous importance since the studies show that veterinarians can have a significant influence on the farmers’ attitudes regarding AMR and AMU practices [73,74].

Within the livestock sector, one of the biggest threats to animal health and welfare, which is also considered as the most common disease that causes huge economic losses in the dairy industry, is bovine mastitis [75]. The etiological agents include a variety of Gram-positive and Gram-negative bacteria [76]. The improvement of biosecurity should be a general measure for prevention of mastitis prevalence; however, currently, antimicrobial treatment remains the main solution to improve animal health and welfare [77], whereby antibiotics are mostly given without the identification of the causative pathogen [78]. Previous research suggests that the most commonly used antibiotics in mastitis therapy in Serbia were penicillin, streptomycin, gentamicin, tetracycline, cephalexin, sulfonamides, and enrofloxacin [79,80]. Furthermore, according to recent data [81,82], the most common mastitis pathogens in dairy cows from Serbia were resistant to penicillin. In the current study, the seven most commonly used antibiotics for cow mastitis therapy were enrofloxacin (17.4%), amoxicillin (14.9%), amoxicillin + clavulanic acid (14.9%), penicillin (10.3%), ceftriaxone (9.3%), tetracycline (8.4%), and gentamicin (7.2%), which is partially consistent with older studies. These data are similar to data from Bangladesh, where amoxicillin, oxytetracycline, ciprofloxacin, and gentamicin were extensively prescribed for large animals, whereas ceftriaxone and penicillin were mainly prescribed just for these animals [83]. Contrary to our findings, the most common antimicrobials prescribed for mastitis in Italy were cephalosporins (30%), followed by potentiated aminopenicillins (11%) and G group penicillin (7%) [55]. It must be highlighted that all antibiotics for mastitis treatment given in our study are considered critically important for human medicine [84,85], which stresses that the ineffective use of antimicrobials has to be regulated. In addition, it would be a valid expectation of the stewardship intervention to achieve a reduction in the use of antibiotics critical for human medicine in both companion animals and dairy cattle [86]. Therefore, we have to allocate our efforts to prudent AMU by arranging resident training for veterinary professionals and focus further research on finding alternatives to antibiotics for this dangerous illness.

### Study Limitations

Certain limitations can be attributed to questionnaire-based studies. Their subjective nature depends on the participants’ opinions and memory. There is also a risk of misinterpretation of questions, which we tried to reduce with as many closed questions as possible. The recorded response rate was 27.3%, which is comparable to similar studies [50,87]. Our result emphasizes the fact that a low response rate of online surveys has been a concern for many researchers in the last few years [88]. Despite this, the relatively low response rate could potentially lead to participation bias. According to the authors’ experience, the possible reason for this could be that many people tend to have more than one email address, often times including an email address that may rarely be checked. During the duration of the study, three reminder emails were sent to the participants in hopes of improving the response rate; however, this did not significantly change the outcome. In future research, some possible incentives in the form of a gift of small economic value or a reward could be considered to improve the response rate. The questionnaire was submitted anonymously, reducing the potential bias of giving only desirable answers. All these elements should be considered in prospective surveys. Although our results cannot be generalized due to the poor response rate, these findings provide important information for evaluating and improving the knowledge and attitudes of farm veterinarians toward AMR and AMU in Serbia.

## 4. Materials and Methods

### 4.1. Ethical Approval

Ethical review and approval of this questionnaire were granted by the University of Novi Sad, Faculty of Agriculture Ethics Committee, via an ethics review application (Ethics approval number: 1047/2/5).

### 4.2. Study Population and Sample Size

According to the data obtained from the Veterinary Chamber of the Republic of Serbia, it is estimated that there are around 700 licensed farm animal veterinarians (September 2021, personal communication). The questionnaire was distributed via the Association of Veterinarians Practitioners of Serbia (AVPS) to their 403 registered members that are currently working in large or mixed veterinary practices, since not all licensed veterinarians are working in practice with farm animals. The questionnaire was distributed using the Google Forms platform. The survey link was sent via emails to the members of the AVPS. The survey was available online for 3 months between 14 May and 14 August 2021. A total of 110 respondents completed the questionnaire, resulting in a response rate of 27.3%.

### 4.3. Study Design—The Questionnaire

The questionnaire used in the study was designed with the intention to collect data on the knowledge and attitudes of farm animal veterinarians on the subjects of AMR and AMU during the period May to August 2021.

The survey was developed after a comprehensive review of the related literature and consultation among the members of research team. The questionnaire was created using a combination of original questions and questions from various surveys [36,37,44,70], with the modifications necessary to ensure the correct answers to questions and claims. It was pretested on 15 veterinarians to receive feedback about understandability and clarity of the questions, and data of the pilot study were not included in the final analysis. The questionnaire was adjusted as per feedback from the piloted sample. It was face-validated via consultation with expert colleagues in the field and was also objectively validated for comprehensibility and clarity. The entire questionnaire took about 10 min to complete.

The full questionnaire is provided in Supplementary File S1 (“Questionnaire on farm animal veterinarians’ knowledge on and attitudes toward antimicrobial resistance and antimicrobial use”).

The questionnaire consisted of 28 questions across four sections aimed at collecting data on demographics, as well as knowledge and attitudes of farm animal veterinarians toward AMR, AMU, and AMS, with a section focused on mastitis therapy. The four sections were designated as sociodemographic data, significance of antimicrobial resistance, rational use of antibiotics, and knowledge of antimicrobial resistance. The first section consisted of six questions regarding demographic data, level of education, and career choices, including age, gender, workplace, the highest level of education, years in practice and average monthly workload based on the number of patients. The second section with 10 questions was focused on participants’ views on the significance of AMR and AMS. The goal was to assess whether the participants kept up to date with this issue and their attitudes toward it in their daily practice. The third section had eight questions about participants’ prescribing habits, factors influencing them, and the rational use of antimicrobials. In the fourth section, the respondents were presented with eight statements with five different levels of agreement (completely agree, somewhat agree, neither agree nor disagree, somewhat disagree, and completely disagree). The statements were used to assess the attitudes and knowledge of participants regarding AMR. Additionally, this section had questions about the most used antibiotics in cow mastitis therapy and the factors influencing decision making when prescribing this therapy.

### 4.4. Data Analysis

The results were extracted from Google Forms to Microsoft Excel. Data were checked for errors, consistency, and uniformity. Statsoft Statistica 12.5 (Tulsa, OK, USA) was used to process the data by means of univariate and multivariate statistical methods. Specifically, the obtained responses were analyzed by descriptive statistics and analysis of response frequency. Furthermore, multivariate correspondence analysis (MCA) was performed in order to better evaluate the complexity of the data matrix structure.

MCA is specially designed for the evaluation of relationships among categorical variables, whether nominal or ordinal. It is a dimension reduction technique, thus enabling data analysts to have better insight into model variability by graphical representation of evaluated variables in the space defined by lower number of dimensions (usually two or three). The calculated dimensions describe a lower proportion of dataset variability (often termed as inertia), but reveal a better structure of the dataset model. In this research, MCA was applied to the dataset represented by a matrix with dimensions of 110 × 75, thus containing a total of 8250 inputs (Supplementary File S2).

## 5. Conclusions

The assessed attitudes and knowledge of Serbian farm veterinarians toward AMR and AMU highlight their mostly reflective attitudes, acknowledging the issue as very important. Veterinarians are trying to educate themselves on AMR; however, there are certain gaps in their knowledge, mainly regarding AMR etiology, personal responsibility when prescribing antibiotics in therapy, use of antibiotics in prophylactic and metaphylactic therapies, and prescribing antibiotics without clear indication (AST tests). The latter, however, seems to be influenced by factors somewhat out of veterinarians’ control, i.e., mainly the cost and the lack of available tests. Most veterinarians also realized the effect that AMU and rational prescription have on the AMR issue, and they were willing to use AMU guidelines to help them in their everyday work, making guidelines for prudent AMU in cattle production systems extremely needed. There was dissatisfaction regarding the number of available local guidelines and the use of antibiotics by unqualified people. Moreover, veterinarians had very positive attitudes toward the possibilities of alternatives to antibiotics.

Unfortunately, the respondents showed a substandard understanding of AMS, making further education and promotion of AMS concepts one of the priorities in our fight for the reduction in AMU and AMR. Additional surveys should be conducted to continuously check the status of our findings.

## Figures and Tables

**Table 1:** Sociodemographic data.

Variable	Response	Frequency (*n* = 110)	Percentage (%)
Gender	Male Female	92 18	83.6 16.4
Age group	25–34 years old	28	25.5
	35–44 years old 45–54 years old 55–64 years old >65 years old	47 19 15 1	42.7 17.3 13.6 0.9
Level of education	Doctor of veterinary medicine	76	69.1
	Master of veterinary medicine	6	5.5
	Doctor of medical sciences—veterinary medicine	14	12.7
	Doctor of veterinary medicine—specialist	14	12.7
Type of employment	Private institution State institution	84 26	76.4 23.6
Number of years working in practice	0–5	17	15.5
	6–15	56	50.9
	>15	37	33.6

**Table 2:** Attitudes toward AMR.

Variable	Response	Frequency (*n* = 110)	Percentage (%)
Received any education on the rational use of antimicrobials or AMR in the last 3 years	Yes No Do not remember	80 29 1	72.7 26.4 0.9
Used domestic or foreign guidelines when prescribing antibiotic therapy	Often Moderately Rarely Never There are no good guidelines	32 39 25 6 8	29.1 35.5 22.7 5.5 7.2
Thought there is a need for more local guidelines for AMU	Yes No Do not know	97 7 6	88.2 6.4 5.4
Encountered ineffective antibiotic therapy for bacterial infections	Daily Weekly Monthly Rarely	5 28 31 44 2	4.5 25.5 28.2 40.0 1.8
	Never		
Heard of antimicrobial stewardship	Yes No	34 76	30.9 69.1
Had protocols for prescribing antibiotics in their practice	Yes No	22 88	20.0 80.0
Did not have protocols for AMU but thought they should have them	Yes No	96 14	87.3 12.7
Kept records of AMU in their practice	Yes No	79 31	71.8 28.2
Prescribed antibiotics outside of indications for their usage	Often Moderately Rarely Never	12 28 47 23	10.9 25.5 42.7 20.9
To which extent does the use of antibiotics by unqualified people negatively impact AMR	There is no impact There is a moderate impact There is a significant impact	1 8 101	0.9 7.3 91.8
Conducted antibiograms (AST tests) routinely	Yes No	54 56	49.1 50.9

**Table 3:** The veterinarians’ opinion on the potential effect of AMS guidelines on specified subjects.

Variable	Frequency (*n* = 110)	Percentage (%)
Increase in responsible prescribing of antibiotics	67	35.6
Reduction in resistant bacteria in humans	49	26.1
Reduction in resistant bacteria in animals	51	27.1
The situation would not significantly change	21	11.2

**Table 4:** The veterinarians’ opinion on potential exposure routes of humans to resistant bacteria.

Variable	Frequency (*n* = 110)	Percentage (%)
Animal products	68	38.9
All of the above	35	20.0
Contact with animals	33	18.9
Contact with other people	17	9.7
Environment	12	6.8
Plants	10	5.7

**Table 5:** The impact of various sectors affecting the development and spread of AMR.

Item	Farm Hygiene	Rational AB Prescribing	Application of AB Therapy by Animal Owners
	Frequency (*n* = 110) Percentage (%)	Frequency (*n* = 110) Percentage (%)	Frequency (*n* = 110) Percentage (%)
Great impact	47 42.7	77 70.0	79 71.8
Medium impact	32 29.1	25 22.7	18 16.4
Small impact	26 23.6	5 4.6	9 8.2
No impact	5 4.6	3 2.7	4 3.6

**Table 6:** Alternatives to antibiotics.

Variable	Frequency (*n* = 110)	Percentage (%)
Probiotics	87	20.5
Vaccines	80	18.9
Prebiotics	62	14.6
Feed enzymes	46	10.9
Immunostimulants	44	10.4
Antimicrobial peptides	24	5.7
Synbiotics	24	5.7
Bacteriocins	21	5.0
Phytocomponents	20	4.7
Phage therapy	9	2.1
Nanoparticles	7	1.7

**Table 7:** Antibiotic therapy influences on the development of AMR.

Item	Great Impact	Medium Impact	Small Impact
	Frequency (*n* = 110) Percentage (%)	Frequency (*n* = 110) Percentage (%)	Frequency (*n* = 110) Percentage (%)
Excessive use of AB	103 93.6	5 4.6	2 1.8
AB use without clear indications (antibiograms)	81 73.6	21 19.1	8 7.3
Wrong therapy length	74 67.3	30 27.3	6 5.4
Low therapy dosage of AB	69 62.7	32 29.1	9 8.2

**Table 8:** Reasons for prescribing antibiotics without a clear indication.

Variable	Frequency (*n* = 110)	Percentage (%)
Animal owners’ financial situation/cost of laboratory tests	67	23.8
The lack of quick diagnostic tests	61	21.6
Pressure from animal owners	47	16.7
Prescribing habits	38	13.5
The lack of clear guidelines for certain diseases	35	12.4
Insufficient education of veterinarians	34	12.1

**Table 9:** Factors that influence AB prescription.

Item	Very Important	Moderately Important	Slightly Important	Not Important
	Frequency (*n* = 110) Percentage (%)	Frequency (*n* = 110) Percentage (%)	Frequency (*n* = 110) Percentage (%)	Frequency (*n* = 110) Percentage (%)
Clinical symptoms	78 70.9	23 20.9	6 5.4	3 2.7
Antibiograms	72 65.4	21 19.1	9 8.2	8 7.3
Milk withholding period for drugs	69 62.7	24 21.8	12 10.9	5 4.5
Concern over AMR spread among animals	62 56.4	34 30.9	10 9.1	4 3.6
Anamnesis	62 56.4	29 26.4	13 11.8	6 5.4
Concern over AMR spread among people	57 51.8	29 26.4	15 13.6	9 8.2
Therapy cost	55 50.0	36 32.7	8 7.3	11 10.0
AB availability	52 47.3	35 31.8	16 14.5	7 6.4
Good practice guidelines	47 42.7	39 35.4	16 14.5	8 7.3
Expectations from animal owners	46 41.8	27 24.5	19 17.3	18 16.4
Expectations from colleagues	20 18.2	31 28.2	28 25.4	31 28.2

**Table 10:** Prescribing habits for antibiotics.

Variable	Frequency (*n* = 110)	Percentage (%)
Exclusively for therapy	106	77.4
For prophylaxis	16	11.7
For metaphylaxis	15	10.9

**Table 11:** Attitudes toward AMR.

Item	Completely Agree	Partially Agree	Neither Agree nor Disagree	Slightly Disagree	Disagree
	Frequency (*n* = 110) Percentage (%)	Frequency (*n* = 110) Percentage (%)	Frequency (*n* = 110) Percentage (%)	Frequency (*n* = 110) Percentage (%)	Frequency (*n* = 110) Percentage (%)
AMR is an important problem in both human and veterinary medicine	102 92.7	4 3.7	2 1.8	2 1.8	0 0
AMR will become much worse in the near future if we do not do something about it now	99 90.0	9 8.2	1 0.9	1 0.9	0 0
Over-the-counter antibiotics should be prohibited	87 79.1	12 10.9	5 4.5	3 2.7	3 2.7
I am open to using alternatives to antibiotics if they are proven to be successful in practice	85 77.3	18 16.4	3 2.7	3 2.7	1 0.9
Uncontrolled use of antibiotics in farm animals is an important cause of resistance to bacterial infections in humans	78 70.9	23 20.9	7 6.4	2 1.8	0 0
The antibiotics I prescribe contribute to the problem of antimicrobial resistance	51 46.4	32 29.1	21 19.1	4 3.6	2 1.8
There is insufficient information on the direct effect of antibiotic use in animals with the development of antimicrobial resistance in humans	51 46.4	37 33.6	12 10.9	4 3.6	6 5.4
AMR is mainly a problem in hospital settings	35 31.8	32 29.1	18 16.4	6 5.4	19 17.3

**Table 12:** The most frequently used antibiotics in cow mastitis therapy.

Variable	Frequency (*n* = 110)	Percentage (%)
Enrofloxacin	56	17.4
Amoxicillin	48	14.9
Amoxicillin + clavulanic acid	48	14.9
Penicillin	33	10.3
Ceftriaxone	30	9.3
Tetracycline	27	8.4
Gentamicin	23	7.2
Trimethoprim + sulfamethoxazole	14	4.4
Cloxacillin	11	3.4
Neomycin	9	2.8
Streptomycin	8	2.5
Ampicillin	5	1.6
Erythromycin	4	1.2
Lincomycin	3	0.9
Novobiocin	2	0.6

**Table 13:** Prescribing habits for cow mastitis therapy.

Variable	Frequency (*n* = 110)	Percentage (%)
Experience and knowledge of clinical symptoms	91	60.6
Exclusively diagnostic tests (antibiograms)	25	16.7
Milk withholding period for antimicrobials	31	20.7
Guidelines (foreign and domestic)	3	2.0

## Data Availability

The data used to support the findings of this study are available in the manuscript or its Supplementary Materials.

## Supplementary Materials

The following are available online at https://www.mdpi.com/article/10.3390/antibiotics11010064/s1: Supplementary File S1: Questionnaire on farm animal veterinarians’ knowledge on and attitudes toward antimicrobial resistance and antimicrobial use; Supplementary File S2: Dataset containing responses collected by questionnaire.

## References

[1] Palma E., Tilocca B., Roncada P. (2020). Antimicrobial resistance in veterinary medicine: An overview. Int. J. Mol. Sci..

[2] Collignon P.C., Conly J.M., Andremont A., McEwen S.A., Aidara-Kane A., Agerso Y., Andremont A., Collignon P., Conly J., tHe World Health Organization Advisory Group, Bogotá Meeting on Integrated Surveillance of Antimicrobial Resistance (WHO-AGISAR) (2016). World Health Organization ranking of antimicrobials according to their importance in human medicine: A critical step for developing risk management strategies to control antimicrobial resistance from food animal production. Clin. Infect. Dis..

[3] Klein E.Y., Van Boeckel T.P., Martinez E.M., Pant S., Gandra S., Levin S.A., Goossens H., Laxminarayan R. (2018). Global increase and geographic convergence in antibiotic consumption between 2000 and 2015. Proc. Natl. Acad. Sci. USA.

[4] Perreten V., Kadlec K., Schwarz S., Grönlund Andersson U., Finn M., Greko C., Moodley A., Kania S.A., Frank L.A., Bemis D.A. (2010). Clonal spread of methicillin-resistant *Staphylococcus pseudintermedius* in Europe and North America: An international multicentre study. J. Antimicrob. Chemother..

[5] Abraham S., Wong H.S., Turnidge J., Johnson J.R., Trott D.J. (2014). Carbapenemase-Producing bacteria in companion animals: A public health concern on the horizon. J. Antimicrob. Chemother..

[6] Williams A., Christley R.M., McKane S.A., Roberts V.L.H., Clegg P.D., Williams N.J. (2013). Antimicrobial resistance changes in enteric *Escherichia coli* of horses during hospitalisation: Resistance profiling of isolates. Vet. J..

[7] Rubin J.E., Pitout J.D. (2014). Extended-Spectrum β-lactamase, carbapenemase and AmpC producing Enterobacteriaceae in companion animals. Vet. Microbiol..

[8] Chantziaras I., Boyen F., Callens B., Dewulf J. (2013). Correlation between veterinary antimicrobial use and antimicrobial resistance in food-producing animals: A report on seven countries. J. Antimicrob. Chemother..

[9] Loeffler A., McCarthy A., Lloyd D.H., Musilová E., Pfeiffer D.U., Lindsay J.A. (2013). Whole-Genome comparison of meticillin-resistant *Staphylococcus aureus* CC22 SCCmecIV from people and their in-contact pets. Vet. Dermatol..

[10] Lloyd D.H., Page S.W., Aarestrup F.M., Schwarz S., Shen J., Cavaco L. (2018). Antimicrobial stewardship in veterinary medicine. Microbiol. Spectr..

[11] Graham D.W., Bergeron G., Bourassa M.W., Dickson J., Gomes F., Howe A., Kahn L.H., Morley P.S., Scott H.M., Simjee S. (2019). Complexities in understanding antimicrobial resistance across domesticated animal, human, and environmental systems. Ann. N. Y. Acad. Sci..

[12] Collignon P.J., McEwen S.A. (2019). One health-its importance in helping to better control antimicrobial resistance. Trop. Med. Infect. Dis..

[13] Laxminarayan R., Duse A., Wattal C., Zaidi A.K.M., Wertheim H.F.L., Sumpradit N., Vlieghe E., Hara G.L., Gould I.M., Goossens H. (2013). Antibiotic resistance—The need for global solutions. Lancet Infect. Dis..

[14] Cantas L., Suer K. (2014). The important bacterial zoonoses in “one health” concept. Front. Public Health.

[15] van den Bogaard A.E., Stobberingh E.E. (2000). Epidemiology of resistance to antibiotics. Links between animals and humans. Int. J. Antimicrob. Agents.

[16] Locke H., Meldrum H. (2011). Use and misuse of antimicrobials. Vet. Rec..

[17] Mahboob A., Altaf I.U.K. (2018). Misuse of antimicrobials. Pak. J. Chest Med..

[18] European Medicines Agency Sales of Veterinary Antimicrobial Agents in 31 European Countries in 2018. https://www.ema.europa.eu/en/documents/report/sales-veterinary-antimicrobial-agents-31-european-countries-2018-trends-2010-2018-tenth-esvac-report_en.pdf.

[19] Economou V., Gousia P. (2015). Agriculture and food animals as a source of antimicrobial-resistant bacteria. Infect Drug Resist..

[20] Van Boeckel T.P., Glennon E.E., Chen D., Gilbert M., Robinson T.P., Grenfell B.T., Levin S.A., Bonhoeffer S., Laxminarayan R. (2017). Reducing antimicrobial use in food animals. Science.

[21] Dyar O.J., Huttner B., Schouten J., Pulcini C. (2017). What is antimicrobial stewardship?. Clin. Microbiol. Infect..

[22] Sanders P., Vanderhaeghen W., Fertner M., Fuchs K., Obritzhauser W., Agunos A., Carson C., Borck Høg B., Dalhoff Andersen V., Chauvin C. (2020). Monitoring of farm-level antimicrobial use to guide stewardship: Overview of existing systems and analysis of key components and processes. Front. Vet. Sci..

[23] Krömker V., Leimbach S. (2017). Mastitis treatment—Reduction in antibiotic usage in dairy cows. Reprod. Domest. Anim..

[24] More S.J. (2020). European perspectives on efforts to reduce antimicrobial usage in food animal production. Ir. Vet. J..

[25] Ferreira J.P., Staerk K. (2017). Antimicrobial resistance and antimicrobial use animal monitoring policies in Europe: Where are we?. J. Public Health Policy.

[26] Van Boeckel T.P., Brower C., Gilbert M., Grenfell B.T., Levin S.A., Robinson T.P., Teillant A., Laxminarayan R. (2015). Global trends in antimicrobial use in food animals. Proc. Natl. Acad. Sci. USA.

[27] Babra C., Tiwari J.G., Pier G., Thein T.H., Sunagar R., Sundareshan S., Isloor S., Hegde N.R., de Wet S., Deighton M. (2013). The persistence of biofilm-associated antibiotic resistance of Staphylococcus aureus isolated from clinical bovine mastitis cases in Australia. Folia Microbiol..

[28] Magouras I., Carmo L.P., Stärk K.D.C., Schüpbach-Regula G. (2017). Antimicrobial usage and -resistance in livestock: Where should we focus?. Front. Vet. Sci..

[29] Tomas A., Pavlović N., Stilinović N., Horvat O., Paut-Kusturica M., Dugandžija T., Tomić Z., Sabo A. (2021). Increase and change in the pattern of antibiotic use in Serbia (2010–2019). Antibiotics.

[30] Robertson J., Iwamoto K., Hoxha I., Ghazaryan L., Abilova V., Cvijanovic A., Pyshnik H., Darakhvelidze M., Makalkina L., Jakupi A. (2019). Antimicrobial medicines consumption in eastern Europeand central Asia—An updated cross-national study and assessment of QuantitativeMetrics for policy action. Front. Pharmacol..

[31] Sl. glasnik RS 30/2010 Law on Medicines and Medical Devices of Serbia. https://www.paragraf.rs/propisi/zakon_o_lekovima_i_medicinskim_sredstvima.html.

[32] Medicines and Medical Devices Agency of Serbia (ALIMS) PROMET VETERINARSKIH LEKOVA 2017–2018. https://www.alims.gov.rs/ciril/files/2021/12/PROMETVETERINARSKIHLEKOVA2017–2018.pdf.

[33] Medicines and Medical Devices Agency of Serbia (ALIMS) Marketing and Consumption of Medicinal Products. https://www.alims.gov.rs/latin/veterinarski-lekovi/promet-i-potrosnja-veterinarskih-lekova/.

[34] European Union (EU) Commission Implementing Decision (EU) 2020/1729 of 17 November 2020 on the Monitoring and Reporting of Antimicrobial Resistance in Zoonotic and Commensal Bacteria and Repealing Implementing Decision 2013/652/EU (Notified under Document C(2020) 7894) (Only the English Version Is Authentic). https://eur-lex.europa.eu/legal-content/en/TXT/?uri=CELEX%3A32020D1729.

[35] Kovacevic Z., Blagojevic B., Suran J., Horvat O. (2020). Mapping knowledge and comprehension of antimicrobial stewardship and biosecurity among veterinary students. PLoS ONE.

[36] Wangmo K., Dorji T., Pokhrel N., Dorji T., Dorji J., Tenzin T. (2021). Knowledge, attitude, and practice on antibiotic use and antibiotic resistance among the veterinarians and para-veterinarians in Bhutan. PLoS ONE.

[37] Taylor D.D., Martin J.N., Morley P.S., Belk K.E., White A.E., Scallan Walter E.J. (2020). Survey of production animal veterinarians’ prescription practices, factors influencing antimicrobial drug use, and perceptions of and attitudes toward antimicrobial resistance. J. Am. Vet. Med. Assoc..

[38] Weese J.S. (2006). Investigation of antimicrobial use and the impact of antimicrobial use guidelines in a small animal veterinary teaching hospital: 1995–2004. J. Am. Vet. Med. Assoc..

[39] Raasch S., Collineau L., Postma M., Backhans A., Sjölund M., Belloc C., Emanuelson U., Beilage E.G., Stärk K., Dewulf J. (2020). Effectiveness of alternative measures to reduce antimicrobial usage in pig production in four European countries. Porc. Health Manag..

[40] Adekanye U.O., Ekiri A.B., Galipó E., Muhammad A.B., Mateus A., La Ragione R.M., Wakawa A., Armson B., Mijten E., Alafiatayo R. (2020). Knowledge, attitudes and practices of veterinarians towards antimicrobial resistance and stewardship in Nigeria. Antibiotics.

[41] Madubuike Umunna A., Oluwatosin Ajoke K. (2017). Veterinarians’ perception, knowledge and practices of antibiotic stewardship in enugu state southeast, Nigeria. Not. Sci. Biol..

[42] WHO Global Action Plan on Antimicrobial Resistance. https://www.who.int/publications/i/item/9789241509763.

[43] Ministry of Health National Antimicrobial Resistance Control Program for 2019–2023. http://www.pravno-informacioni-sistem.rs/SlGlasnikPortal/prilozi/1.html&doctype=reg&abc=cba&eli=true&eliActId=427789&regactid=427789.

[44] Hardefeldt L.Y., Gilkerson J.R., Billman-Jacobe H., Stevenson M.A., Thursky K., Bailey K.E., Browning G.F. (2018). Barriers to and enablers of implementing antimicrobial stewardship programs in veterinary practices. J. Vet. Intern. Med..

[45] Espinosa-Gongora C., Jessen L.R., Dyar O.J., Bousquet-Melou A., González-Zorn B., Pulcini C., Re G., Schwarz S., Timofte D., Toutain P.-L. (2021). Towards a better and harmonized education in antimicrobial stewardship in European veterinary curricula. Antibiotics.

[46] Llanos-Soto S.G., Vezeau N., Wemette M., Bulut E., Greiner Safi A., Moroni P., Shapiro M.A., Ivanek R. (2021). Survey of perceptions and attitudes of an international group of veterinarians regarding antibiotic use and resistance on dairy cattle farms. Prev. Vet. Med..

[47] Ekakoro J.E. (2018). Antimicrobial use practices of veterinary clinicians at a veterinary teaching hospital in the United States. Vet. Anim. Sci..

[48] Wayne A., McCarthy R., Lindenmayer J. (2011). Therapeutic antibiotic use patterns in dogs: Observations from a veterinary teaching hospital. J. Small Anim. Pract..

[49] Gozdzielewska L., King C., Flowers P., Mellor D., Dunlop P., Price L. (2020). Scoping review of approaches for improving antimicrobial stewardship in livestock farmers and veterinarians. Prev. Vet. Med..

[50] Scherpenzeel C.G.M., Santman-Berends I.M.G.A., Lam T.J.G.M. (2018). Veterinarians’ attitudes toward antimicrobial use and selective dry cow treatment in the Netherlands. J. Dairy Sci..

[51] Nelson D.W., Moore J.E., Rao J.R. (2019). Antimicrobial resistance (AMR): Significance to food quality and safety. Food Qual. Saf..

[52] Aubry-Damon H., Grenet K., Sall-Ndiaye P., Che D., Cordeiro E., Bougnoux M.-E., Rigaud E., Le Strat Y., Lemanissier V., Armand-Lefèvre L. (2004). Antimicrobial resistance in commensal flora of pig farmers. Emerg. Infect. Dis. J..

[53] Damborg P., Olsen K.E., Møller Nielsen E., Guardabassi L. (2004). Occurrence of *Campylobacter jejuni* in pets living with human patients infected with *C. jejuni*. J. Clin. Microbiol..

[54] Pozza G., Pinto A., Crovato S., Mascarello G., Bano L., Dacasto M., Battisti A., Bartoli B., Ravarotto L., Marangon S. (2020). Antimicrobial use and antimicrobial resistance: Standpoint and prescribing behaviour of Italian cattle and pig veterinarians. Ital. J. Anim. Sci..

[55] Busani L., Graziani C., Binkin N., Franco A., Di Egidio A., Battisti A. (2004). Survey of the knowledge, attitudes and practice of Italian beef and dairy cattle veterinarians concerning the use of antibiotics. Vet. Rec..

[56] World Health Organization (WHO) No Time to Wait: Securing the Future from Drug-Resistant Infections. https://www.who.int/docs/default-source/documents/no-time-to-wait-securing-the-future-from-drug-resistant-infections-en.pdfsfvrsn=5b424d7_6.

[57] Singh S.B., Young K., Silver L.L. (2017). What is an “ideal” antibiotic? Discovery challenges and path forward. Biochem. Pharmacol..

[58] Boyd N.K., Teng C., Frei C.R. (2021). Brief overview of approaches and challenges in new antibiotic development: A focus on drug repurposing. Front. Cell. Infect. Microbiol..

[59] Kumar M., Sarma D.K., Shubham S., Kumawat M., Verma V., Nina P.B., JP D., Kumar S., Singh B., Tiwari R.R. (2021). Futuristic non-antibiotic therapies to combat antibiotic resistance: A review. Front. Microbiol..

[60] Sirichokchatchawan W., Apiwatsiri P., Pupa P., Saenkankam I., Khine N.O., Lekagul A., Lugsomya K., Hampson D.J., Prapasarakul N. (2021). Reducing the risk of transmission of critical antimicrobial resistance determinants from contaminated pork products to humans in south-east Asia. Front. Microbiol..

[61] Servia-Dopazo M., Taracido-Trunk M., Figueiras A. (2021). Non-Clinical factors determining the prescription of antibiotics by veterinarians: A systematic review. Antibiotics.

[62] De Briyne N., Atkinson J., Pokludová L., Borriello S.P., Price S. (2013). Factors influencing antibiotic prescribing habits and use of sensitivity testing amongst veterinarians in Europe. Vet. Rec..

[63] Chipangura J.K., Eagar H., Kgoete M., Abernethy D., Naidoo V. (2017). An investigation of antimicrobial usage patterns by small animal veterinarians in South Africa. Prev. Vet. Med..

[64] Kumar V., Gupta J., Meena H. (2019). Assessment of awareness about antibiotic resistance and practices followed by veterinarians for judicious prescription of antibiotics: An exploratory study in eastern haryana region of India. Trop. Anim. Health Prod..

[65] Bourély C., Fortané N., Calavas D., Leblond A., Gay É. (2018). Why do veterinarians ask for antimicrobial susceptibility testing? A qualitative study exploring determinants and evaluating the impact of antibiotic reduction policy. Prev. Vet. Med..

[66] Hardy B. (2002). The issue of antibiotic use in the livestock industry: What have we learned?. Anim. Biotechnol..

[67] Ungemach F.R., Müller-Bahrdt D., Abraham G. (2006). Guidelines for prudent use of antimicrobials and their implications on antibiotic usage in veterinary medicine. Int. J. Med. Microbiol..

[68] Jorritsma R., Van der Heide A., Van Geijlswijk I.M. (2021). Survey of veterinarians in the Netherlands on antimicrobial use for surgical prophylaxis in dairy practice. J. Dairy Sci..

[69] Firouzabadi D., Mahmoudi L. (2020). Knowledge, attitude, and practice of health care workers towards antibiotic resistance and antimicrobial stewardship programmes: A cross-sectional study. J. Eval. Clin. Pract..

[70] Zhuo A., Labbate M., Norris J.M., Gilbert G.L., Ward M.P., Bajorek B.V., Degeling C., Rowbotham S.J., Dawson A., Nguyen K.A. (2018). Opportunities and challenges to improving antibiotic prescribing practices through a One Health approach: Results of a comparative survey of doctors, dentists and veterinarians in Australia. BMJ Open.

[71] Odoi A., Samuels R., Carter C.N., Smith J. (2021). Antibiotic prescription practices and opinions regarding antimicrobial resistance among veterinarians in Kentucky, USA. PLoS ONE.

[72] Speksnijder D.C., Jaarsma A.D.C., van der Gugten A.C., Verheij T.J.M., Wagenaar J.A. (2015). Determinants associated with veterinary antimicrobial prescribing in farm animals in the Netherlands: A qualitative study. Zoonoses Public Health.

[73] Cattaneo A.A., Wilson R., Doohan D., LeJeune J.T. (2009). Bovine veterinarians’ knowledge, beliefs, and practices regarding antibiotic resistance on Ohio dairy farms. J. Dairy Sci..

[74] McDougall S., Compton C., Botha N. (2017). Factors influencing antimicrobial prescribing by veterinarians and usage by dairy farmers in New Zealand. N. Z. Vet. J..

[75] Gomes F., Henriques M. (2016). Control of bovine mastitis: Old and recent therapeutic approaches. Curr. Microbiol..

[76] Cervinkova D., Vlkova H., Borodacova I., Makovcova J., Babak V., Lorencova A., Vrtkova I., Marosevic D., Jaglic Z. (2013). Prevalence of mastitis pathogens in milk from clinically healthy cows. Vet. Med..

[77] Cheng W.N., Han S.G. (2020). Bovine mastitis: Risk factors, therapeutic strategies, and alternative treatments—A review. Asian-Australas. J. Anim. Sci..

[78] Oliveira L., Ruegg P.L. (2014). Treatments of clinical mastitis occurring in cows on 51 large dairy herds in Wisconsin. J. Dairy Sci..

[79] Vakanjac S., Pavlović V., Magaš V., Pavlović M., Đurić M., Maletić M., Nedić S., Sočo I. (2013). Investigations of efficacy of intramammary applied antimicrobials and glucocorticosteroides in the treatment of subclinical and clinical mastitis in cows. Vet. Glas..

[80] Anđelković J., Radonjić V. (2017). Usage of intramammary antimicrobial veterinary medicinal products in the republic of Serbia from 2011 to 2014. Serb. J. Exp. Clin. Res..

[81] Kovačević Z., Radinović M., Čabarkapa I., Kladar N., Božin B. (2021). Natural agents against bovine mastitis pathogens. Antibiotics.

[82] Kovačević Z., Kladar N., Čabarkapa I., Radinović M., Maletić M., Erdeljan M., Božin B. (2021). New perspective of *Origanum vulgare L.* and *Satureja montana L.* essential oils as bovine mastitis treatment alternatives. Antibiotics.

[83] Rahman M.S., Rafa N. (2021). Common barriers, attitudes, and practices of veterinary practitioners regarding antimicrobial resistance and stewardship in Chattogram, Bangladesh. Open Vet. Sci..

[84] World Health Organization (WHO) Critically Important Antimicrobials for Human Medicine. https://www.who.int/publications/i/item/9789241515528.

[85] OIE List of Antimicrobial Agents of Veterinary Importance. https://www.oie.int/app/uploads/2021/03/a-oie-list-antimicrobials-june2019.pdf.

[86] Acharya K.R., Brankston G., Soucy J.-P.R., Cohen A., Hulth A., Löfmark S., Davidovitch N., Ellen M., Fisman D.N., Moran-Gilad J. (2021). Evaluation of an OPEN stewardship generated feedback intervention to improve antibiotic prescribing among primary care veterinarians in Ontario, Canada and Israel: Protocol for evaluating usability and an interrupted time-series analysis. BMJ Open.

[87] Postma M., Speksnijder D.C., Jaarsma A.D., Verheij T.J., Wagenaar J.A., Dewulf J. (2016). Opinions of veterinarians on antimicrobial use in farm animals in Flanders and the Netherlands. Vet. Rec.

[88] Fan W., Yan Z. (2010). Factors affecting response rates of the web survey: A systematic review. Comput. Hum. Behav..

