# Supplementary material for: Farm Animal Veterinarians’ Knowledge and Attitudes toward Antimicrobial Resistance and Antimicrobial Use in the Republic of Serbia
Source: Antibiotics (Basel). 2022 Jan 5;11(1):64. doi: 10.3390/antibiotics11010064 (PMC8773276; doi:10.3390/antibiotics11010064)
Supplement: Supplementary file 1 [file antibiotics-11-00064-s001.zip › antibiotics-1522259-Supplementary File S1.pdf]

QUESTIONNAIRE ON FARM ANIMAL VETERINARIANS' KNOWLEDGE AND ATTITUDES TOWARDS ANTIMICROBIAL RESISTANCE AND ANTIMICROBIAL USE

I Sociodemographic data

1. Year of birth? (write) ___________

2. Gender (circle): a) female

   b) male

3. Do you work in (circle):

   a) a state institution

   b) a private institution

4. Your highest level of education (circle):

a) Doctor of Veterinary Medicine

b) Masters of Veterinary Medicine

c) Doctor of Medical Sciences - Veterinary Medicine

d) Doctor of Veterinary Medicine - Specialist

5. Number of years you worked in practice (circle):

   a) 0 - 5

   b) 6 - 15

   c) > 15

6. What is the average number of patients you examine on a monthly basis? (write): ______

II Significance of antimicrobial resistance

1. In the last three years, have you attended any education on the rational use of antibiotics or antibacterial resistance? (circle):

a) Yes

b) No

c) I educated myself on antimicrobial resistance using available scientific sources of information

d) I do not remember

2. Do you use domestic or foreign scientific guidelines when prescribing antibiotics in your daily work? (Circle):

Often / moderately / rarely or never / there are no good guidelines

3. Would you like to have more local guidelines for the rational use of antibiotics? (Circle):

Yes / No / I do not know

4. Have you encountered bacterial infections for which most antibiotics have been ineffective in your daily work? (circle):

Daily / Weekly / Monthly / Rarely / Never

5. To what extent do you think these factors influence the development and spread of antibiotic resistance? (Circle):

Hygiene on farms	Insignificant / small impact / medium impact / large impact
Rational prescribing of antibiotics by a veterinarian	Insignificant / small impact / medium impact / large impact
Rational use of antibiotics in farm animals	Insignificant / small impact / medium impact / large impact
Rational use of antibiotics by patients	Insignificant / small impact / medium impact / large impact

6. What are the sources of transmission of resistant bacteria to humans (multiple answers):

a) contact with other people

b) animal products (milk, meat, eggs)

c) contact with animals

d) plants

e) environment

f) all of the above

7. Are you familiar with the term Antimicrobial stewardship and its' principles?

YES / NO

8. **Antimicrobial stewardship (AMS) is defined as an ongoing effort by a health service organisation to optimise antimicrobial use among patients 'to improve patient outcomes, ensure cost-effective therapy and reduce adverse sequelae of antimicrobial use (including antimicrobial resistance)**. Taking this into consideration do you think that Antimicrobial Stewardship Guidelines and Regulations can contribute to (multiple responses):

a) responsible prescribing of antibiotics

b) reducing the occurrence of antibiotic-resistant bacteria in humans

c) reducing the occurrence of antibiotic-resistant bacteria in animals

e) it will not significantly change the situation

9. Are you familiar with the listed alternatives to antibiotics? (Circle)

1. vaccines
2. probiotics
3. prebiotics
4. synbiotics
5. antimicrobial peptides
6. phage therapy
7. bacteriocins
8. phytocomponents
9. immunostimulants
10. nanoparticles
11. feed enzymes

10. What is the level of contribution of the following factors to the resistance of bacteria to antibiotics (circle):

Excessive use of antibiotics	low / medium / high
Low doses of antibiotics	low / medium / high
Prescribing antibiotics in therapy without appropriate tests (antibiogram)	low / medium / high
Inadequate length of antibiotic therapy (too short or too long)	low / medium / high

III Rational use of antibiotics

1. Do you have regulations for prescribing antibiotics in your practice / on the farm?

YES / NO

2. If you do not have these regulations, do you think they should exist?

YES / NO

3. Do you keep records of the amount of antibiotics used in your practice?

YES / NO

4. Do you routinely conduct an antibiogram in case of antibiotic therapy? Circle:

YES / NO

5. What are the reasons for prescribing antibiotics without a clear indication? Multiple answers:

a) insufficient education of veterinarians

b) pressure from the owner of the animal (patient)

c) prescribing habits

d) lack of rapid diagnostic tests

e) financial situation of animal owners / costs of microbiological tests

f) lack of clear guidelines for some conditions and diseases

6. How often do you prescribe antibiotics for indications outside the instructions for the use of the drug?

often / moderately / rarely or never

7. Significance of factors in decision making when prescribing antibiotics

Care for the spread of antimicrobial resistance in humans	Very significant / Moderately significant / Partly significant / Insignificant
Care for the spread of antimicrobial resistance in animals	Very significant / Moderately significant / Partly significant / Insignificant
Anamnesis	Very significant / Moderately significant / Partly significant / Insignificant
Clinical signs	Very significant / Moderately significant / Partly significant / Insignificant
Availability of antibiotics	Very significant / Moderately significant / Partly significant / Insignificant
Antibiogram	Very significant / Moderately significant / Partly significant / Insignificant
Treatment costs	Very significant / Moderately significant / Partly significant / Insignificant
Milk withdrawal period	Very significant / Moderately significant / Partly significant / Insignificant
Expectations by animal owners	Very significant / Moderately significant / Partly significant / Insignificant
Expectations from colleagues	Very significant / Moderately significant / Partly significant / Insignificant
Good practice guidelines	Very significant / Moderately significant / Partly significant / Insignificant

8. When do you prescribe antibiotics (multiple answers possible):

a) as metaphylaxis

b) as prophylaxis

c) exclusively for therapeutic purposes

9. To what extent do you think the use of antibiotics by unqualified persons affects the appearance of resistance?

There is no impact / there is a moderate impact / there is a significant impact

IV Knowledge of antimicrobial resistance

1. On the scale shown next to each statement, circle the extent to which you agree with it. One answer per claim.

		Completely agree	Partially agree	Neither agree nor disagree	Slightly disagree	Do not agree
1.	AMR is an important problem in both human and veterinary medicine.	5	4	3	2	1
2.	Bacterial resistance to antibiotics will become a far more significant problem in the clinic in the near future if we do not take appropriate measures now.	5	4	3	2	1
3.	The antibiotics I prescribe contribute to the problem of antimicrobial resistance.	5	4	3	2	1
4.	Uncontrolled use of antibiotics in farm animals is an important cause of resistance to bacterial infections in humans.	5	4	3	2	1
5.	There is insufficient information on the direct effect of antibiotic use in animals with the development of antimicrobial resistance in humans.	5	4	3	2	1
6.	Over-the-counter antibiotics should be prohibited.	5	4	3	2	1

7.	The problem of antibiotic resistance of bacteria is mainly a problem in hospital settings.	5	4	3	2	1
8.	I am open to using alternatives to antibiotics if they are proven to be successful in practice.	5	4	3	2	1

Cow mastitis antibiotic therapy

1. Circle the 3 antibiotics that you most often prescribe in the treatment of mastitis in cows:

1. Amoxicillin
2. Ampicillin
3. Ceftriaxone
4. Enrofloxacin
5. Erythromycin
6. Gentamicin
7. Lincomycin
8. Neomycin
9. Penicillin
10. Streptomycin
11. Tetracycline
12. Amoxicillin + Clavulanic acid
13. Novobiocin
14. Trimethoprim + Sulfamethoxazole
15. Cloxacillin

2. When prescribing antibiotics for the treatment of mastitis in cows, I am mostly guided by (circle):

a) personal professional experience and knowledge of the clinical signs

b) regulations (domestic and foreign)
c) exclusively diagnostic analysis (antibiogram)

d) the duration of the milk withdrawal period
